# Elastic SCAD as a novel penalization method for SVM classification tasks in high-dimensional data

**DOI:** 10.1186/1471-2105-12-138

**Published:** 2011-05-09

**Authors:** Natalia Becker, Grischa Toedt, Peter Lichter, Axel Benner

**Affiliations:** 1 German Cancer Research Center (DKFZ), Division Molecular Genetics, INF 280, 69120 Heidelberg, Germany; 2 German Cancer Research Center (DKFZ), Division Biostatistics, INF 280, 69120 Heidelberg, Germany

## Abstract

**Background:**

Classification and variable selection play an important role in knowledge discovery in high-dimensional data. Although Support Vector Machine (SVM) algorithms are among the most powerful classification and prediction methods with a wide range of scientific applications, the SVM does not include automatic feature selection and therefore a number of feature selection procedures have been developed. Regularisation approaches extend SVM to a feature selection method in a flexible way using penalty functions like LASSO, SCAD and Elastic Net.

We propose a novel penalty function for SVM classification tasks, Elastic SCAD, a combination of SCAD and ridge penalties which overcomes the limitations of each penalty alone.

Since SVM models are extremely sensitive to the choice of tuning parameters, we adopted an interval search algorithm, which in comparison to a fixed grid search finds rapidly and more precisely a global optimal solution.

**Results:**

Feature selection methods with combined penalties (Elastic Net and Elastic SCAD SVMs) are more robust to a change of the model complexity than methods using single penalties. Our simulation study showed that Elastic SCAD SVM outperformed LASSO (*L*_1_) and SCAD SVMs. Moreover, Elastic SCAD SVM provided sparser classifiers in terms of median number of features selected than Elastic Net SVM and often better predicted than Elastic Net in terms of misclassification error.

Finally, we applied the penalization methods described above on four publicly available breast cancer data sets. Elastic SCAD SVM was the only method providing robust classifiers in sparse and non-sparse situations.

**Conclusions:**

The proposed Elastic SCAD SVM algorithm provides the advantages of the SCAD penalty and at the same time avoids sparsity limitations for non-sparse data. We were first to demonstrate that the integration of the interval search algorithm and penalized SVM classification techniques provides fast solutions on the optimization of tuning parameters.

The penalized SVM classification algorithms as well as fixed grid and interval search for finding appropriate tuning parameters were implemented in our freely available R package 'penalizedSVM'.

We conclude that the Elastic SCAD SVM is a flexible and robust tool for classification and feature selection tasks for high-dimensional data such as microarray data sets.

## Background

Classification and prediction methods play important roles in data analysis for a wide range of applications. Frequently, classification is performed on high-dimensional data, where the number of features is much larger compared to the number of samples ('large p small n' problem) [1]. In those cases, classification by Support Vector Machines (SVM), originally developed by Vapnik [2], is one of the most powerful techniques. The SVM classifier aims to separate the samples from different classes by a hyperplane with largest margin.

Often we do not only require a prediction rule but also need to identify relevant components of the classifier. Thus, it would be useful to combine feature selection methods with SVM classification. Feature selection methods aim at finding the features most relevant for prediction. In this context, the objective of feature selection is three-fold: (i) improving the prediction performance of the predictors, (ii) providing faster and more cost-effective predictors, and (iii) gaining a deeper insight into the underlying processes that generated the data.

Three main groups of feature selection methods exist: filter, wrapper and embedded methods [1,3-6]. Filter methods simply rank individual features by independently assigning a score to each feature. These methods ignore redundancy and inevitably fail in situations where only a combination of features is predictive. Also, if there is a pre-set limit on the number of features to be chosen (e.g. top 10 features), this limit is arbitrary and may not include all informative features. Because of these drawbacks, the filter methods are not included in this work.

Connecting filtering with a prediction procedure, wrapper methods wrap feature selection around a particular learning algorithm. Thereby, prediction performance of a given learning method assesses only the usefulness of subsets of variables. After a subset with lowest prediction error is estimated, the final model with reduced number of features is built [5]. However, wrapper methods have the drawback of high computational load, making them less applicable when the dimensionality increases. Wrapper methods also share the arbitrariness of filter methods in feature selection.

The third group of feature selection procedures are embedded methods, which perform feature selection within learning classifiers to achieve better computational efficiency and better performance than wrapper methods. The embedded methods are less computationally expensive and less prone to overfitting than the wrappers [7].

Guyon [1] proposed the recursive feature elimination (RFE) method, which belongs to the wrapper methods. RFE iteratively keeps a subset of features which are ranked by their contribution to the classifier. This approach is computationally expensive and selecting features based only on their ranks may not derive acceptable prediction rules.

An alternative to SVM with RFE is to use penalized SVM with appropriate penalty functions. Penalized SVM belongs to embedded methods and provides an automatic feature selection. The investigation of the widely used family of penalization functions such as LASSO, SCAD, Elastic Net [8-10] and a novel proposed penalty Elastic SCAD in combination with SVM classification, is the objective of the paper. The ridge penalty [4] corresponds to the ordinary SVM, which does not provide any feature selection, is used as reference with respect to prediction accuracy.

Although feature selection methods can be applied to any high-dimensional data, we illustrate the use of these methods on microarray gene expression data due to their relevance in cancer research. Data from microarray experiments are usually stored as large matrices of expression levels of genes in rows and different experimental conditions in columns. Microarray technology allows to screen thousand of genes simultaneously. Detailed reviews on the technology and statistical methods often used in microarray analyses are presented in [11-13].

Since SVM is extremely sensitive to the choice of tuning parameters, the search for optimal parameters becomes an essential part of the classification algorithm [14]. The problem of choosing appropriate tuning parameters is discussed and an interval search technique from Froehlich and Zell [15] is proposed to use for SVM classification.

In this paper, we investigate the behaviour of feature selection SVM classifier techniques including commonly used penalization methods together with a novel penalization method, the Elastic SCAD. We compare them to SVM classification with and without recursive feature elimination (RFE [1]) for situations of 'large p small n' problems.

The RFE SVM is chosen as as a state-of-the-art representative of feature selection methods in applications [16,17].

A simulation study is designed to investigate the behaviour of different penalization approaches. Publicly available microarray data sets are chosen for illustration purposes as applications on real high-dimensional data.

## Methods

### Support Vector Machines

Suppose a training data set with input data vector **x***_i _*∈ ℝ*^p ^*and corresponding class labels *y_i _*∈ {-1, 1}, *i *= 1,..., *n *is given. The SVM finds a maximal margin hyperplane such that it maximises the distance between classes. A linear hyperplane can always perfectly separate *n *samples in *n *+ 1 dimensions. Since we can assume that high-dimensional data with *p *≫ *n *is generally linear separable [6], increasing complexity by using non-linear kernels is usually not needed. Thus, we use a linear SVM model throughout the paper.

The linear SVM separates classes by a linear boundary(1)

where **w **= (*w*_1_, *w*_2_,..., *w_p_*) is a unique vector of coefficients of the hyperplane with ||**w**||_2 _= 1 and *b *denotes the intercept of the hyperplane. We use '·' to denote the inner product operator. The class assignment for a test data vector **x**_test _∈ **R***^p ^*is given by *y*_test _= sign [*f *(**x**_test_)].

#### Soft margin SVM

Soft margin SVM allows some data points to be on the wrong side of the margin. To account for erroneous decisions, slack variables *ξ_i _*≥ 0, *i *= 1,..., *n *are defined as the distance between a misclassified data point and the corresponding margin. For data points on the correct side of the margin *ξ_i _*= 0, for data points inside the margin 0 <*ξ_i _*≤ 1 and for misclassified data points *ξ_i _*> 1. The sum of non-zero *ξ_i _*is penalized with a cost parameter *C *and then added to the optimisation function penalty in the minimisation problem:(2)

The optimisation problem (2) is called the *soft margin SVM*. The cost parameter *C *is a data dependent tuning parameter that controls the balance between minimizing the coefficients of the hyperplane and correct classification of the training data set. *C *is often chosen by cross validation. Problem (2) can be solved by using convex optimisation techniques, namely by the method of Lagrange multipliers [4]. Convex optimisation techniques provide a unique solution for hyperplane parameters **w **and *b*(3)

where *α_i _*≥ 0, *i *= 1,..., *n *are Lagrange multipliers. The data points with positive *α_i_*, are called *support vectors *(SVs). All data points lying on the correct side of their margin have *α_i _*= 0. Thus, they do not have any impact on the hyperplane, and we can rewrite Eq. (3) as(4)

where the set of indices of the support vectors S is determined by *S *:= {*i *: *α_i _*> 0}.

The coefficient  can be calculated from  for any *i *with *α_i _*> 0. In praxis, an average of all solutions for  is used for numerical stability.

### SVM as a penalization method

Hastie et al. [4] showed that the SVM optimisation problem is equivalent to a penalization problem which has the "*loss and penalty*" form(5)

where the loss term is described by a sum of the hinge loss functions *l *(*y_i_*, *f *(*x_i_*)) = [1 - *y_i _f *(**x***_i_*)]_+ _= max(1 - *y_i _f *(**x***_i_*), 0) for each sample vector **x***_i_*, *i *= 1,..., *n*. The penalty term is denoted as pen*_λ _*(**w**) and can have different forms:

### Ridge penalty

The penalty term for ordinary SVM uses the *L*_2 _norm:(6)

The *L*_2 _penalty shrinks the coefficients to control their variance. However, the ridge penalty provides no shrinkage of the coefficients to zero and hence no feature selection is performed.

### LASSO

The use of a *L*_1 _penalization function is originally proposed by Tibshirani [8] for generalized linear models. The technique for parameter estimation with constraints is called LASSO ('least absolute shrinkage and selection operator'). Later, Bradley [18] adapted the *L*_1_-regularisation to SVM. Then, the penalty term has the form(7)

As a result of singularity of the *L*_1 _penalty function, *L*_1 _SVM automatically selects features by shrinking coefficients of the hyperplane to zero.

However, the *L*_1 _norm penalty has two limitations. First, the number of selected features is bounded by the number of samples. Second, it tends to select only one feature from a group of correlated features and drops the others.

Fung and Mangasarian [19] have published a fast *L*_1 _SVM modification, the Newton Linear Programming Support Vector Machine (NLPSVM), which we use in our analyses.

### Smoothly clipped absolute deviation penalty (SCAD)

The SCAD penalty is a non-convex penalty function first proposed by Fan and Li [20]. Later, Zhang et al. [10] combined the SVM technique with the SCAD penalty for feature selection. The SCAD penalty function for a single coefficient *w_j _*is defined as

where *w_j _*, *j *= 1,..., *p *are the coefficients defining the hyperplane and *a *> 2 and λ > 0 are tuning parameters. Fan and Li [21] showed that SCAD prediction is not sensitive to selection of the tuning parameter *a*. Their suggested value *a *= 3.7 is therefore used in our analyses.

The penalty term for SCAD SVM has the form

The SCAD penalty corresponds to a quadratic spline function with knots λ at and *a*λ. For small coefficients *w_j_*, *j *= 1,..., *p*, SCAD yields the same behaviour as *L*_1_. For large coefficients, however, SCAD applies a constant penalty, in contrast to *L*_1_. This reduces the estimation bias. Furthermore, the SCAD penalty holds better theoretical properties than the *L*_1 _penalty [21].

### Elastic Net

To overcome the limitations of LASSO, Zou and Hastie [9] proposed a linear combination of *L*_1 _and *L*_2 _penalties which they called *Elastic Net*:(8)

The Elastic Net penalty provides automatic feature selection similar to *L*_1_, but is no longer bounded by the sample size. Moreover, at the same time this penalty manages to select highly correlated features (*grouping effect*). Increasing *λ*_1 _reduces the number of features of the classifier whereas for large *λ*_2 _one observes better control of the grouping effect. Wang [22] adapted the Elastic Net penalty to SVM classification problems. Therefore, the Elastic Net SVM optimisation problem can be written as

where *λ*_1_, *λ*_2 _≥ 0 are the corresponding tuning parameters.

### Elastic SCAD

Fan and Li [21] demonstrated the advantages of the SCAD penalty over the *L*_1 _penalty. However, using the SCAD penalty might be too strict in selecting features for non-sparse data. A modification of the SCAD penalty analogously to Elastic Net could keep the advantages of the SCAD penalty, and, at the same time, avoid too restrictive sparsity limitations for non-sparse data.

We therefore propose a combination of the SCAD and the *L*_2 _penalties. The new penalty term has the form

*λ*_1_, *λ*_2 _≥ 0 are the tuning parameters. We expect that the Elastic SCAD will improve the SCAD method for less sparse data. According to the nature of the SCAD and *L*_2 _penalties, the Elastic SCAD should show good prediction accuracy for both, sparse and non-sparse data.

It can be shown that the combined penalty provides sparsity, continuity, and asymptotic normality when the tuning parameter for the ridge penalty converges to zero, i.e. *λ*_2 _→ 0. The asymptotic normality and sparsity of Elastic SCAD leads to the oracle property in the sense of Fan and Li [21].

The Elastic SCAD SVM optimisation problem has the form(9)

where *λ*_1_, *λ*_2 _≥ 0 are the tuning parameters.

### Elastic SCAD SVM: Algorithm

By solving Eq. (9) the same problems as for SCAD SVM occur: the hinge loss function is not differentiable at zero and the SCAD penalty is not convex in **w**. The Elastic SCAD SVM objective function can be locally approximated by a quadratic function and the minimisation problem can be solved iteratively similar to the SCAD approach [10,21].

For simplicity, we rename the SCAD penalty from  to . Accordingly, the first-order derivative of the penalty is denoted by . Denote the penalized objective function in Eq. (9) by

For each *i *(with respect to the fact that ) the loss term can be split according to

Given an initial value (*b*_0_; **w**_0_) close to the minimum of *A*(*b*, **w**), we consider the following local quadratic approximations:

When *w*_*j*0 _is close to zero, set ; otherwise use the approximation for the SCAD penalty

where due to symmetrical nature of the SCAD penalty |*w_j_*| is used instead of *w_j_*.

It can be shown that both approximations and their original functions have the same gradient at the point (*b*_0_, **w**_0_). Therefore, the solution of the local quadratic function corresponds approximately to the solution of the original problem.

The local quadratic approximation of *A*(*b*, **w**) has the form

By minimisation of *A*(*b*, **w**) with respect to **w **and *b*, terms without optimisation parameters **w **and *b *can be dropped (due to derivatives of constants):

To write the equations in matrix form we define:

Moreover, we define the matrix *X *= [**1**, **x**_1_,..., **x***_p_*], where **1 **is the vector of 1s with length n and **x***_j _*is the *j*th input vector. Set

Minimizing *A *(*b*, **w**) is then equivalent to minimizing the quadratic function(10)

The solution to Eq. (10) satisfies the linear equation system(11)

The Elastic SCAD SVM can be implemented by the following iterative algorithm.

**Step 1 **Set *k *= 1 and specify the initial value (*b*^(1)^, **w**^(1)^) by standard *L*_2 _SVM according to Zhang et al. [10].

**Step 2 **Store the solution of the *k*th iteration: (*b*_0_, **w**_0_) = (*b*^(*k*)^, **w**^(*k*)^).

**Step 3 **Minimize Ã (*b*, **w**) by solving Eq. (11), and denote the solution as (*b*^(*k*+1)^, **w**^(*k*+1)^).

**Step 4 **Let *k *= *k *+ 1. Go to step 2 until convergence.

If elements  are close to zero, for instance, smaller than 10^-4^, then the *j*th variable is considered to be redundant and in the next step will be removed from the model. The algorithm stops after convergence of (*b*^(*k*)^, **w**^(*k*)^).

### Choosing tuning parameters

All SVM problems with or without feature selection use one or two tuning parameters which balance the trade-off between data fit and model complexity. Since these parameters are data dependent, finding optimal tuning parameters is part of the classification task.

### Fixed grid search

Tuning parameters are usually determined by a grid search. The grid search method calculates a target value, e.g. the misclassification rate, at each point over a fixed grid of parameter values. This method may offer some protection against local minima but it is not very efficient. The density of the grid plays a critical role in finding global optima. For very sparse grids, it is very likely to find local optimal points. By increasing the density of the grid, the computation cost increases rapidly with no guaranty of finding global optima. The major disadvantage of the fixed grid approach lies in the systematic check of the misclassification rates in each point of the grid. There is no possibility to skip redundant points or to add new ones.

When more parameters are included in the model, the computation complexity is increased. Thus, the fixed grid search is only suitable for tuning of very few parameters.

### Interval search

Froehlich and Zell [15] suggested an efficient algorithm of finding a *global *optimum on the tuning parameter space using a method called EPSGO (Efficient Parameter Selection via Global Optimisation).

The main idea of the EPSGO algorithm is to treat the search for an optimal tuning parameter as a global optimisation problem. For that purpose, the Gaussian Process model is learned from the points in the parameter space which have been already visited. Thereby, training and testing of the GP is very efficient in comparison to the calculation of the original SVM models. New points in the parameter space are sampled by using the expected improvement criterion as described in the EGO algorithm [23], which avoids stacking in local minima. The stopping criteria of the EPSGO algorithm is either convergence of the algorithm or no change of the optimum during the last ten iterations.

### Stratified cross validation

Using *k*-fold cross validation, the data set is randomly split into *k *disjoint parts of roughly equal size, usually *k *= 5 or *k *= 10. In addition, the data is often split in a way that each fold contains approximately the same distribution of class labels as the whole data set, denoted by *stratified *cross validation. For each subset, one fits the model using the other *k *- 1 parts and calculates the prediction error of the selected *k*th part of the data.

The case *k *= *n *is called *leave one out cross validation *(LOO CV). The choice of *k *determines a trade-off between bias and variance of the prediction error. Kohavi [24] showed that ten-fold stratified cross validation showed better performance in terms of bias and variance compared to 10 <*k *<*n*. Hastie et al. [4] recommended to perform five- or ten-fold cross validation as a good compromise between variance and bias. We used both, five- and ten-fold stratified cross validation for simulation study and real applications, respectively.

In the next two sections the application of penalized SVM classification methods are compared. We used simulated and publicly available data to investigate the behaviour of different feature selection SVMs. For all comparisons the R pack-ages "penalizedSVM" [25] and "e1071" [26] were used which are freely available from the CRAN http://cran.r-project.org/, R version 2.10.1. The R package "e1071" is a wrapper for the well-known LIBSVM software [27]. We used five- and ten-fold stratified cross validation in combination with interval search for tuning parameters as described above.

## Results and Discussion

### Simulation study

#### Simulation design

A comprehensive simulation study evaluating the performance of four feature selection SVM classifiers, *L*_1 _SVM, SCAD SVM, Elastic Net SVM and Elastic SCAD SVM, was performed. We used the ordinary *L*_2 _SVM algorithm with a liner kernel as a reference for prediction accuracy.

Two independent data sets are simulated: a training set for building the classifier and a test set for estimating of the prediction errors of classifiers. First, the training data is generated, and the optimal tuning parameters are found using five-fold stratified cross validation according to the interval search approach [15]. Then, the classification hyperplane is computed using the estimated tuning parameters. Finally, application of the classification rule to the test data provides the prediction characteristics such as misclassification error, sensitivity and specificity.

Training and test input data are represented by a data matrix **X **= {**x***_i_*}, *i *= 1,..., *n*, where **x***_i _*∈ ℝ*^p ^*describes feature patterns for the *i*th sample. The input data **X **follows a multivariate normal distribution with mean *μ *and covariance matrix Σ. The class labels **Y **= {*Y_i_*}, *i *= 1,..., *n *are generated according to a logistic regression model

where *β *= {*β *_1_,..., *β *_p_} is a vector of coefficients of a classifier and *u_i _*are realisations of a variable following a *U *0[1] distribution.

In our simulations the percentage of relevant features varies between 1% and 20%. Coefficients *β _j_*, *j *= 1,..., *p *are always defined as

with equal numbers of positive and negative coefficients. The intersect *β *_0 _is set to zero.

We also consider to have 'clumps' of correlated features. The clumpy dependency is supposed to describe the most common type of dependency in microarray studies [28]. We define "clumps" of correlated features as blocks of one relevant and four redundant features with a covariance matrix Σ*^(*k*)^, where *k *is the number of the current block. The diagonal elements of Σ*^(*k*) ^for each block are equal to one and the off-diagonal elements are equal to *ρ *= 0.8. In total, we design five blocks of correlated features and therefore the covariance matrix has the form

where

Due to clumping blocks, the vector of *β *has a more complex form

with

where *r *denotes the number of relevant features. Using correlated blocks we investigate the ability of selecting correlated features, the so called *grouping effect*.

Optimal tuning parameters are found by an interval search in tuning parameter space using five fold cross validation. We select a large tuning parameter interval to be certain not to stick in local optima. The tuning parameter space for *L*_1 _and SCAD SVM is one-dimensional with *λ*_1 _∈ [*λ*_1,*min*_, *λ*_1,*max*_]. Elastic SCAD has two tuning parameters *λ*_1_, *λ*_2 _∈ [*λ_l, min_*, *λ*_*l, max*_], *l *= 1, 2. Elastic Net applies LARS paths. for fixed *λ*_2 _a *λ*_1 _path is calculated and the optimal *λ*_1 _is identified (for details refer to [17]). Thus, the optimal pair of parameter  for Elastic Net was found in the two-dimensional space ℝ × [*λ*_*l, min*_, *λ*_*l, max*_] We set the search interval for both parameters to [*λ*_*l, min*_, *λ*_*l, max*_] = [2^-10^, 2^-10^], *l *= 1, 2.

The performance of classifiers is characterised by the Youden index. The Youden index describes as equally weighted sum of true positive results ("sensitivity") and false positive results ("1 - specificity"):

The maximal Youden index is one, when the true positive rate is one and the false positive rate is zero. For a random classifier the expected Youden index is zero. The sensitivity and specificity have equal weights in this index. Most often the costs and con-sequences of true positives and false positives will differ greatly. Therefore, Gu and Pepe [29] recommend reporting the two measures separately. For our simulated data, we consider the Youden index to be an appropriate indicator for feature selection methods performance, since we weight errors equally.

It is worth to mention, that for discrete classier the Youden index and the area under the curve (AUC) provide the same message due to their linear relationship. According to Greiner et al. [30], if there is only one point in the ROC plot, the ROC curve is estimated by connecting the three points. the point corresponds to the classifier, the (0, 0) and (1, 1) edges of the plot. Then geometrically, the estimated AUC corresponds to the average of estimated sensitivity and specificity. Thus, the Youden index and the AUC have a linear relationship. AUC = (sensitivity + specificity)/2 = (Youden index +1)/2. Optimizing the AUC will lead to the same results as optimizing the Youden index when dealing with discrete classifiers. Nevertheless, for real data application, the AUC values are presented in a separate column due to higher level of familiarity in bioinformatics.

Finally, the misclassification rate, size of the classifiers and frequencies of the selected features within 100 simulation runs are computed.

#### Simulation results

The performance of the feature selection methods applied to simulated data using *p *= 1000 features and *n *= 500 samples for training and testing is presented in the next section. The percentage of relevant features varies between 1% and 20% in four steps, i.e. r = 10, 50, 100, 200. We assume to have correlated blocks of features as described in the design section. The optimal tuning parameters were chosen as described above. Multiple comparisons in performance measures between the proposed prediction methods and the best method (the MCB test) for each simulation step will be done according to Hsu [31] based on 100 simulation runs. We used a non-inferiority margin of a procedure to distinguish methods with similar performance.

#### Misclassification rate

Table 1 summarises the average misclassification rates depending on the number of relevant features. The numbers in parentheses are the standard errors of the estimates. For very sparse models (10 out of 1000 features are relevant) SCAD showed the lowest misclassification error (18%), followed by Elastic Net and Elastic SCAD (19.4% and 20.8% respectively), where both lie in indifference zone for best methods if the non-inferiority margin was set to Δ = 0.05. For less sparse to non-sparse models (*r *= 50 and *r *= 100) Elastic Net showed the best performance. For *r *= 200 relevant features *L*_1 _and Elastic Net showed nearly the same results (32.9% and 33.1% respectively). The same was observed for SCAD (34.7%) and Elastic SCAD (34.2%). For *r *≥ 50 the misclassification rate was indistinguishable for all feature selection methods with exception of the *L*_1 _SVM. The *L*_2 _SVM classifiers showed larger misclassification errors for sparse models (*r *= 10 and *r *= 50) than all other feature selection methods. For less sparse models differences in misclassification error levelled out.

**Table 1:** Mean misclassification rate of feature selection methods applied to simulated test data

FS method	r = 10	r = 50	r = 100	r = 200
*L*_2 _SVM	34.8_(2.2)_	33.1_(2.0)_	**33.3**_(2.1)_	**32.8**_(1.9)_

*L*_1 _SVM	28.3_(2.8)_	**28.6**_(3.0)_	**32.4**_(2.2)_	**32.9**_(2.1)_
SCAD SVM	**18.0**_(2.2)_	**27.2**_(4.4)_	**35.3**_(3.4)_	**34.7**_(4.1)_
Elastic Net SVM	**19.4**_(2.0)_	**24.7**_(3.0)_	**31.3**_(2.3)_	**33.1**_(2.7)_
Elastic SCAD SVM	**20.8**_(4.5)_	**26.8**_(4.2)_	**33.1**_(2.7)_	**34.2**_(4.1)_

Training and test data with 1000 features and 500 samples were simulated. The number of relative features (r) were increased from r = 10 to r = 200 in four steps. Each simulation step was based on 100 simulations of training and test data. In **bold **- the significant best method(s) according to the MCB test at the family-wise significance level *α *= 0.05 and non-inferiority margin of Δ = 5%.

#### Youden index

The average Youden index for very sparse models (*r *= 10) was considerably high for all feature selection methods: 0.96 for SCAD, 0.95 for Elastic Net, 0.92 for Elastic SCAD, and 0.81 for *L*_1 _SVM (Table 2). By increasing number of informative features, the Elastic Net SVM showed the best Youden index (0.71% - 0.27%) among all feature selection methods, closely followed by the Elastic SCAD SVM (0.67% - 0.27%), both being indistinguishable.

**Table 2:** Average Youden index for classifiers applied to simulated test data

FS method	r = 10	r = 50	r = 100	r = 200
*L*_1 _SVM	0.81_(0.11)_	**0.59**_(0.12)_	0.32_(0.16)_	0.14_(0.10)_
SCAD SVM	**0.96**_(0.06)_	**0.65**_(0.12)_	0.28_(0.12)_	**0.16**_(0.07)_
Elastic Net SVM	**0.95**_(0.04)_	**0.71**_(0.09)_	**0.48**_(0.07)_	**0.27**_(0.05)_
Elastic SCAD SVM	**0.92**_(0.11)_	**0.67**_(0.13)_	**0.42**_(0.09)_	**0.27**_(0.06)_

In **bold **- the significant best method(s) according to the MCB test at the family-wise significance level *α *= 0.05 and non-inferiority margin of Δ = 0.10.

All methods except the L1 SVM provided significantly comparable Youden indexes at the level *α *= 0.05 and a relevant difference Δ = 0.10 for *r *= 10. By increasing model complexity, the Elastic Net SVM showed the best Youden Index among all feature selection methods, closely followed by the Elastic SCAD SVM. Starting from *r *> 100 the is no significant difference between Elastic Net and Elastic SCAD SVMs. With increasing number of relevant features, the Youden index decreases from 0.9 to 0.27 for 'elastic' methods to 0.14 for the *L*_1 _SVM and to 0.16 for the SCAD SVM. respectively.

#### Sparsity of the classifier

The SCAD SVM provided the most sparse classifier (in terms of selecting the smallest number of features) for *r *= 10 and *r *= 50 out of 1000 features (cf. Table 3). It selected 12 and 61 features, respectively. For less sparse models the Elastic Net and the Elastic SCAD SVMs had similar performance, selecting the smallest number of features.

**Table 3:** Median number of features selected

FS method	r = 10	r = 50	r = 100	r = 200
*L*_1 _SVM	141_(56)_	296_(98)_	509_(290)_	789_(223)_
SCAD SVM	**12**_(3)_	**61**_(24)_	593_(382)_	726_(181)_
Elastic Net SVM	38_(25)_	242_(110)_	**355**_(164)_	511_(183)_
Elastic SCAD SVM	24_(19_)	161_(139)_	430_(116)_	**493**_(124)_

In **bold **- median number of features that come closest to the true number of relevant features per simulation scenario, (in parentheses - median absolute deviation); underline - the second best.

#### Selection Frequencies

A frequencies plot for the simulation study is represented in 'Additional file 1 - Frequencies plot'. With increasing number of relevant features (*r*), a decrease of the proportion of true positives (in red) and an increase of the proportion of false positives (in blue) for all feature selection models was observed, respectively. At the same time we observed an increase of the false positives, which are correlated with the true positives (in green) in classifiers.

The percentage of true positives in the classifiers is shown in Table S1 (Additional file 2 -- Tables S1, S2, S3). For *r *= 10 relevant features the Elastic Net SVM found almost all true positives (99.8%), followed by the Elastic SCAD SVM with 97.6%. For *r *= 50 the Elastic SCAD SVM achieved the sparsest solution followed by the *L*_1 _SVM. In less sparse models, the *L*_1 _SVM showed highest true positive rates of 84.5% and 86%.

#### Grouping effect

We further evaluated the ability of feature selection methods to select correlated features of true positives. Although for all scenarios *L*_1 _SVM has found the largest percentage of correlated features, which increases with increasing number of relevant features (23.6 - 62.5%), the level of correlated features is comparable to the level of non-relevant features (Table S2).

Comparing Tables S1, S2 and S3 one can observe that the SCAD and the *L*_1 _SVMs failed to find features highly correlated with true positives more often than with independent false positives. The Elastic Net and the Elastic SCAD SVMs managed to discover correlated features (in green) more often than the independent false positives (in blue), at least for sparse models (*r *= 10 and *r *= 50).

#### The false positive rate

For very sparse models, the false positive rate (FPR) was the smallest for the SCAD SVM, followed by the Elastic Net and the Elastic SCAD SVMs (Table S3). For other less sparse models the Elastic Net SVM selected fewer false positives than the remaining methods. The second best method is the Elastic SCAD SVM.

#### Conclusions

• As expected from theory the SCAD SVM and the *L*_1 _SVM produced classifiers with low prediction error for very sparse situations.

• For less sparse and non-sparse models, the Elastic Net and the Elastic SCAD SVM showed better results than the *L*_1 _and the *L*_2 _SVMs with respect to accuracy, Youden index and sparsity of classifiers.

• The SCAD SVM and the *L*_1 _SVM were not able to find correlated features. The Elastic Net and the Elastic SCAD SVMs found correlated features more frequently than one would expected under random selection. Although the grouping effect strength weakens with increasing number of relevant features, the Elastic Net and Elastic SCAD SVMs still managed the grouping effects.

• In general, the Elastic Net and the Elastic SCAD SVMs showed similar performance. Additionally, the Elastic SCAD SVM provided more sparse classifiers than the Elastic Net SVM.

### Applications

#### NKI breast cancer data set

Two studies on breast cancer from the Netherlands Cancer Institute (NKI) were published by the van't Veer group [32], [33]. In the first paper, a set of 78 lymph node negative patients with pre-selected 4919 clones were used to find a predictor for distant metastases. The classifier was trained and validated on patients who developed distant metastases within five years after surgery and patients being metastasis-free for at least the first five years. The resulting predictor was a 70-gene signature also known as *MammaPrint(R)*. We will use the MammaPrint(R) signature as reference in the analysis of the NKI breast cancer data set. The signature was generated based on gene-wise correlations between the gene expression and metastasis occurrence. The data set was taken from http://www.rii.com/publications/2002/vantveer.html.

In a subsequent validation study, data from 295 patients (which partially included patients from the first study) were used to validate the signature [33]. Among the patients, 151 were lymph node negative and 144 had lymph node positive disease. The pre-processed data containing 4919 clones is available from http://www.rii.com/publications/2002/nejm.html.

After excluding patients being identical to the training set and 10 patients with no metastasis information, 253 patients remained. Among the 253 patients there are 114 lymph node negative and 139 lymph node positive patients.

In this paper, we combined the 78 lymph node negative sample set from the first publication with 114 lymph node negative patients from the validation study. In total, a data set with 192 lymph node negative samples was used. The estimation of classifier performance was computed by a ten-fold stratified cross-validation.

#### Results on NKI breast cancer data set

Table 4 shows the misclassification error, sensitivity, specificity, Youden index and AUC value of four feature selection methods, RFE SVM and standard *L*_2 _SVM based on ten-fold stratified cross validation.

**Table 4:** Summary of classifiers for the NKI data set with distant metastasis as endpoint

FS method	# features	test error(%)	sensitivity(%)	specificity(%)	Youden index	AUC
*L*_2 _SVM	4919 (all)	24	79	68	0.47	0.735
RFE SVM	256	25	83	59	0.42	0.71

MammaPrint(R)	70	37	74	40	0.14	0.57

*L*_1 _SVM	1573	17	84	81	0.65	0.825
SCAD SVM	476	25	84	56	0.39	0.695
Elastic Net SVM	109	25	83	59	0.42	0.71
Elastic SCAD SVM	459	24	84	57	0.41	0.705

Misclassification error, sensitivity, specificity, Youden index and AUC value for four feature selection methods, RFE SVM and standard SVM based on ten-fold stratified cross validation.

RFE SVM was used according to Guyon's approach [1], where at each iteration half of features with lowest ranks are eliminated. To increase the classifier's stability, RFE SVM with five-fold stratified cross validation was repeated five times. According to the average cross validation error the optimal number of features was 2^8 ^= 256. Optimal tuning parameters for penalized SVM methods were found by the interval search on the tuning parameter space as described in the method section using ten-fold stratified cross validation.

The SCAD SVM reduced the number of features from 4919 to 476, *L*_1 _SVM selected 1573 features, Elastic Net 109 features, and the Elastic SCAD had 459 features in the classifier. For the NKI data set the best predictor with respect to misclassification error was *L*_1 _SVM. Elastic Net and Elastic SCAD SVMs provided similar results, followed by SCAD SVM, which was slightly worse.

**Figure 1:**
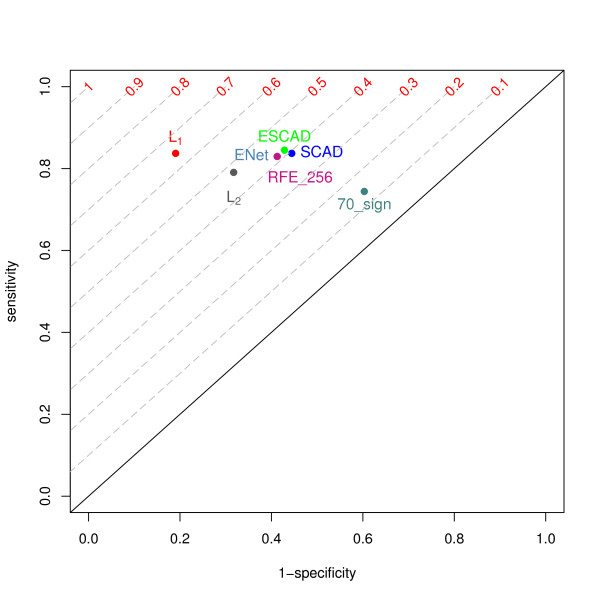
**ROC plot for the NKI breast data set**. The characteristics for the different feature selection methods were derived using ten-fold stratified cross validation. TPR and FPR values are presented as points (x axis: 1- specificity = FPR, y axis. sensitivity = TPR). RFE_256 is RFE SVM with 256 top ranked features, ENet is Elastic Net SVM, ESCAD is Elastic SCAD SVM. '70_sign' stands for the 70-gene signature classifier. Gray dashed lines depict isolines of the Youden index.

The relationship between the true positive rate (TPR, sensitivity) and the false positive rate (FPR, 1-specificity) for each classifier is depicted as a point in the ROC plot (Figure 1). Isolines with constant Youden index are plotted as dashed lines. Taking the Youden index as an additional criterion, one could prioritise *L*_1 _SVM. RFE SVM and both 'elastic' methods lay clustered in the ROC plot with clear distance to the *L*_1 _classifier. The *L*_2 _was placed in-between *L*_1 _and this cluster, being not far from the cluster.

Interestingly, the MammaPrint(R) signature ("70_sign") neither showed good test accuracy nor a reliable sensitivity or specificity. *L*_2 _SVM and the feature selection methods outperformed the published signature.

#### Conclusions

For the two data sets from van't Veer group feature selection methods produced signatures with similar prediction accuracy, but being different in size. *L*_1 _SVM with a non-sparse classifier provided the best sensitivity and specificity, followed by more sparse predictors from Elastic Net SVM and Elastic SCAD SVM.

#### MAQC-II breast cancer data set

This data set is part of the MicroArray Quality Control (MAQC)-II project, which has been designed to investigate numerous data analysis methods and to reach consensus on the "best practices" for development and validation of microarray-based classifiers for clinical and preclinical applications. One biological endpoint is estrogen receptor (ER) status. Out of 230 patients in total, 89 patients have negative ER status and 141 patients positive ER status. A clinical endpoint is pathological complete response (pCR) to preoperative chemotherapy. Among the 230 patients in the data set, 182 patients showed no pCR and 48 had a pCR.

The preprocessed data contains 22283 features and is available from GEO database, accession number GSE20194.

#### Results on MAQC-II breast cancer data set

The feature selection methods SCAD SVM, *L*_1 _SVM, Elastic Net SVM and Elastic SCAD SVM with internal ten-fold stratified cross validation were applied to build classifiers. Additionally, the *L*_2 _SVM and the RFE SVM were used as reference models. To achieve performance measurements ten-fold stratified cross validation was used.

#### pCR prediction

Based on the minimal average misclassification error, the optimal number of features of RFE SVM classifier was obtained to be 2^11 ^= 2048 (Table 5). The penalized SVM methods provided moderately sparse models, Elastic SCAD SVM with 148 features, Elastic Net SVM with 398 features and dense models, *L*_1_, SCAD and RFE SVMs with more than 1000 features.

**Table 5:** Summary of classifiers for the MAQC-II data set with pCR status as endpoint

FS method	# features	test error(%)	sensitivity(%)	specificity(%)	Youden index	AUC
*L*_2 _SVM	22283 (all)	19	32	97	0.25	0.62
RFE SVM	2048	20	27	93	0.20	0.895

*L*_1 _SVM	7299	21	27	93	0.20	0.60
SCAD SVM	1072	21	35	91	0.26	0.63
Elastic Net SVM	398	24	15	91	0.06	0.53
Elastic SCAD SVM	148	15	52	94	0.46	0.73

Misclassification error, sensitivity, specificity, Youden index and AUC value for four feature selection methods, RFE SVM and standard SVM based on ten-fold stratified cross validation.

**Figure 2:**
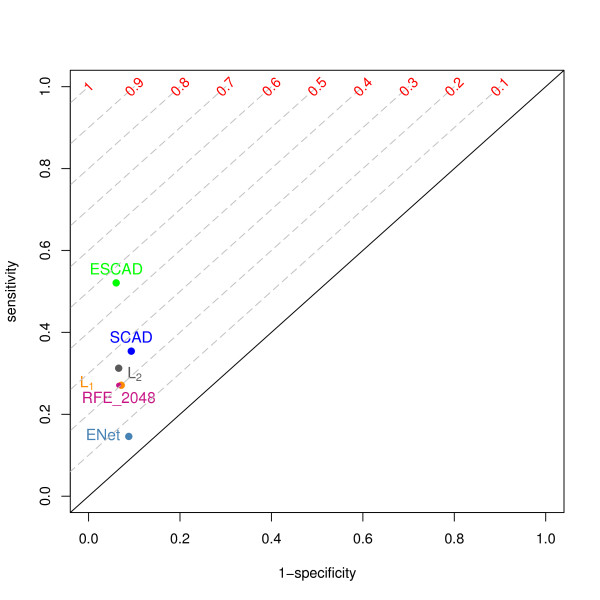
**ROC plot for MAQC-II breast data set with pCR as endpoint**. The characteristics for the different feature selection methods were derived using ten-fold statrifierd cross validation. TPR and FPR values are presented as points (x axis: 1- specificity = FPR, y axis. sensitivity = TPR). RFE_256 is RFE SVM with 1024 top ranked features, ENet is Elastic Net SVM, ESCAD is Elastic SCAD SVM. Gray dashed lines depict isolines of the Youden index.

The misclassification error rate was similar for all methods with the Elastic SCAD classifier showing the lowest error rate of 15%. With nearly equally high specificity (91-94%), we observed large variations in sensitivity of different feature selection methods as shown in the corresponding ROC plot (Figure 2). The Elastic SCAD outperformed all methods with sensitivity of 52%. Interestingly, the Elastic Net showed the smallest sensitivity of 15% resulting in a small Youden index of 0.06.

Overall, Elastic SCAD showed better classification characteristics than other methods. Moreover, the higher specificity of the Elastic SCAD classifier is of clinical importance. The patients that did not respond to the therapy were recognized with higher probability.

#### ER status

We also used the MAQC-II data set to predict the ER status. Here, the *L*_1 _SVM failed to derive a sparse solution, whereas SCAD, Elastic Net and Elastic SCAD SVM classifiers were similar (Table 6). Moreover, Elastic SCAD showed the smallest error rate and highest sensitivity over all methods.

**Table 6:** Summary of classifiers for the MAQC-II data set with ER status as endpoint

FS method	# features	test error(%)	sensitivity(%)	specificity(%)	Youden index	AUC
*L*_2 _SVM	22283 (all)	10	93	84	0.77	0.855
RFE SVM	2048	14	89	81	0.79	0.895

*L*_1 _SVM	860	11	89	88	0.77	0.885
SCAD SVM	32	9	91	91	0.83	0.915
Elastic Net SVM	3	9	93	82	0.75	0.875
Elastic SCAD SVM	59	7	96	88	0.84	0.92

Misclassification error, sensitivity, specificity, Youden index and AUC value for four feature selection methods, RFE SVM and standard SVM without feature selection based on ten-fold stratified cross validation.

**Figure 3:**
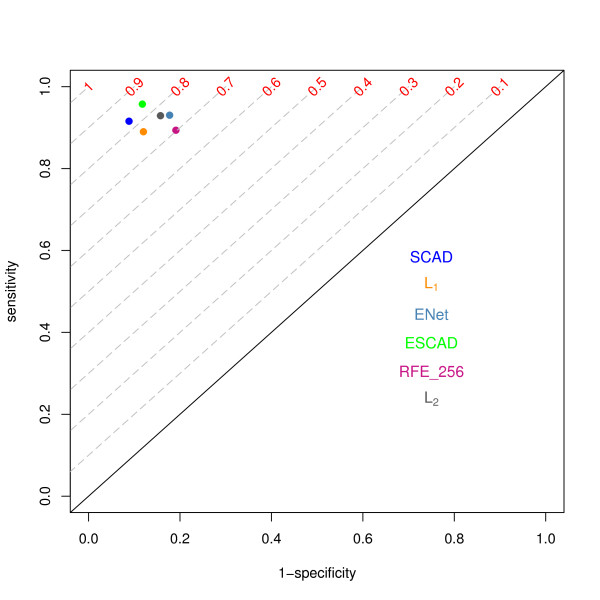
**ROC plot for MAQC-II breast data set with ER as endpoint**. The characteristics for the different feature selection methods were derived using ten-fold stratified cross validation. TPR and FPR values are presented as points (x axis: 1- specificity = FPR, y axis. sensitivity = TPR). RFE_256 is RFE SVM with 1024 top ranked features, ENet is Elastic Net SVM, ESCAD is Elastic SCAD SVM. Gray dashed lines depict isolines of the Youden index.

All classification methods provided small misclassification errors, high sensitivity and high specificity. The ROC plot in Figure 3 demonstrates this performance of predictors. As presented in Table 6 the Elastic Net, SCAD and Elastic SCAD SVMs selected small numbers of features, 3, 32 and 59 out of 22283, respectively. The extreme sparseness of the Elastic Net SVM was paid by lower sensitivity and specificity compared to other methods. The misclassification test error was similar for all methods (7-14%). The Elastic SCAD SVM classifier showed the smallest error rate of 7%.

For this classification task, the sparse classifier Elastic SCAD and SCAD showed the best characteristics.

#### Screening on two additional breast cancer data sets

These data sets were recently analysed and published by Johannes et. al. [34]. The first data set, the Mainz cohort, contains of 154 lymph node-negative, relapse free patients and 46 lymph node-negative patients that suffered a relapse (GEO acession number GSE11121). The relapse is defined as appearance of distant metastasis within five years after the treatment. The second data set, the Rotterdam cohort, represents 286 lymph node-negative breast cancer samples including 107 re-lapse events (GSE2034). Both data sets were generated using the Affymetrix HG-U133A platform, normalized with the same methods and relapse as the primary classification endpoint. We trained the feature selection classifiers on the whole cohort, Mainz data or Rotterdam data, and used the other cohort as an independent validation data set, respectively as presented in Tables 7 and 8.

**Table 7:** Summary of classifiers for Mainz cohort, validated on Rotterdam cohort with relapse as endpoint

FS method	# features	test error(%)	sensitivity(%)	specificity(%)	Youden index	AUC
*L*_2 _SVM	22283 (all)	44	68	48	0.16	0.58
RFE SVM	512	37	38	77	0.16	0.58

*L*_1 _SVM	1861	37	47	72	0.19	0.595
SCAD SVM	915	37	35	80	0.15	0.575
Elastic Net SVM	278	43	51	60	0.12	0.56
Elastic SCAD SVM	2823	37	34	81	0.15	0.575

Misclassification error, sensitivity, specificity, Youden index and AUC value for four feature selection methods, RFE SVM and standard SVM trained on the Mainz cohort and applied to the Rotterdam cohort.

**Table 8:** Summary of classifiers for Rotterdam cohort, validated on Mainz cohort with relapse as endpoint

FS method	# features	test error(%)	sensitivity(%)	specificity(%)	Youden index	AUC
*L*_2 _SVM	22283 (all)	25	11	93	0.04	0.52
RFE SVM	22283 (all)	25	11	93	0.04	0.52

*L*_1 _SVM	8319	28	30	84	0.14	0.57
SCAD SVM	1284	35	41	72	0.13	0.565
Elastic Net SVM	272	28	37	81	0.19	0.595
Elastic SCAD SVM	2074	26	30	87	0.17	0.585

Misclassification error, sensitivity, specificity, Youden index and AUC value for four feature selection methods, RFE SVM and standard SVM trained on the Rotterdam cohort and applied to the Mainz cohort.

We can see that all feature selection methods had lower misclassification test error than the *L*_2 _SVM containing all features for breast cancer data sets. The classifiers perform different for each data set. The Elastic Net SVM had small error rate for the Rotterdam cohort, but failed to classify the Mainz samples adequately. The *L*_2 _SVM classifier including all features had the second best Youden index for the Mainz set, however for Rotterdam data showed the worst Youden index. Using both, the test error and AUC value as a combined measure of sensitivity and the specificity, one would conclude that the *L*_1_, SCAD and Elastic SCAD SVMs provide reasonable and robust solutions with respect to the combined analysis of the two breast cancer data sets.

Altogether, Elastic SCAD seems to provide an overall acceptable compromise for sparse and non-sparse data.

## Conclusions

In high-dimensional prediction tasks, feature selection plays an important role. In this paper, we proposed a novel feature selection method for SVM classification using a combination of two penalties, SCAD and *L*_2_. The commonly used penalty functions *L*_1_, SCAD and Elastic Net were investigated in parallel with the new method on simulated and public data. To address the problem of finding optimal tuning parameters for SVM classification the efficient parameter search algorithm from Froehlich and Zell [15] was implemented.

In almost all cases, the four feature selection classifies outperformed ordinary Support Vector Classification using the *L*_2 _penalty. From the simulation study we concluded that for sufficiently large sample sizes, feature selection methods with combined penalties are more robust to changes of the model complexity than using single penalties alone.

The SCAD SVM followed by the *L*_1 _SVM, as expected, showed very good performance in terms of pre-diction accuracy for very sparse models, but failed for less sparse models. Combined penalty functions in combination with the SVM algorithm, Elastic Net and Elastic SCAD, performed well for sparse and less sparse models.

Comparisons with commonly used penalty functions in the simulation study illustrated that the Elastic SCAD and the Elastic Net SVMs showed similar performance with respect to prediction accuracy. Both 'elastic' methods were able to consider correlation structures in the input data (grouping effect). However, the Elastic SCAD SVM in general provides more sparse classifiers than the Elastic Net SVM.

Finally, applied to publicly available breast cancer data sets, the Elastic SCAD SVM performed very flexible and robust in sparse and non-sparse situations. Results from the simulation study and real data application render Elastic SCAD SVM with automatic feature selection a promising classification method for high-dimensional applications.

## Authors' contributions

NB and AB contributed to the design of the simulation study and to theoretical investigations of problems. NB performed simulations, analyses and wrote the manuscript. GT and AB participated in the preparation of the manuscript. AB and PL supervised the work. All authors read and approved the manuscript.

## Supplementary Material

Additional file 1 **Frequencies plot**. Frequencies of selected features in the classifiers after 100 runs. In x-axis: features, y-axis: frequency of appearing of each features in classifiers after 100 runs. Features: true positives or non-zero (in red), zero features correlated with true positives (in green) and true negatives or zero (in blue). Algorithms from left to right: SCAD SVM, 1-norm (*L*_1_) SVM, Elastic Net SVM and Elastic SCAD SVM. Number of features: from top to bottom from very sparse till non-sparse models, *r*: 10, 50, 100, 200 out of 1000 features are relevant. Click here for file

Additional file 2 **Tables S1, S2, S3**. **Table S1: Mean frequency percentages for non-zero features in the classifier**. Mean frequency percentages for non-zero features in the classifier (true positives) after 100 runs. Standard deviations in parentheses. **Table S2: Mean frequency percentages for zero features, high correlated with non-zero features in the classifier**. Mean frequency percentages for zero features, high correlated with non-zero features in the classifier after 100 runs. Standard deviations in parentheses. **Table S3: Mean frequency percentages for independent non-zero features in the classifier (false positives)**. Mean frequency percentages for independent non-zero features in the classifier (false positives) after 100 runs. Standard deviations in parentheses. Click here for file
