# Supplementary figures and images for: Elastic SCAD as a novel penalization method for SVM classification tasks in high-dimensional data
Source: BMC Bioinformatics. 2011 May 9;12:138. doi: 10.1186/1471-2105-12-138 (PMC3113938; doi:10.1186/1471-2105-12-138)

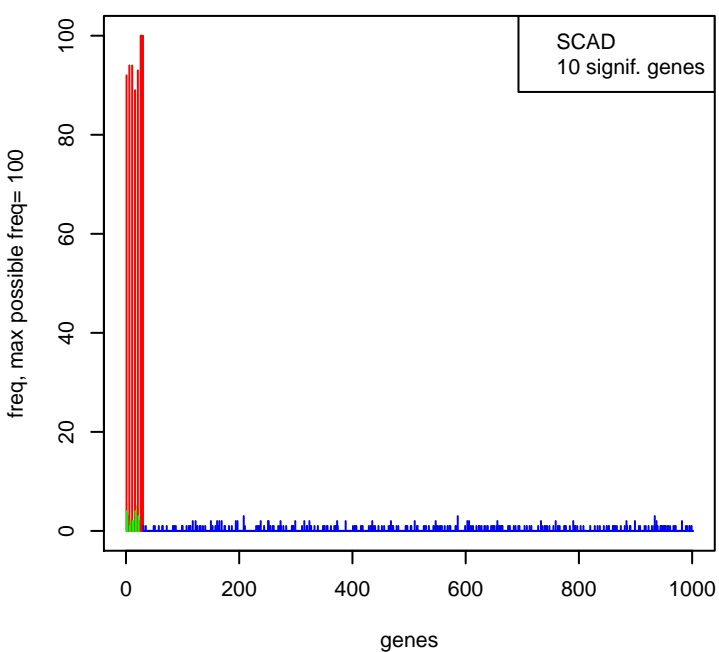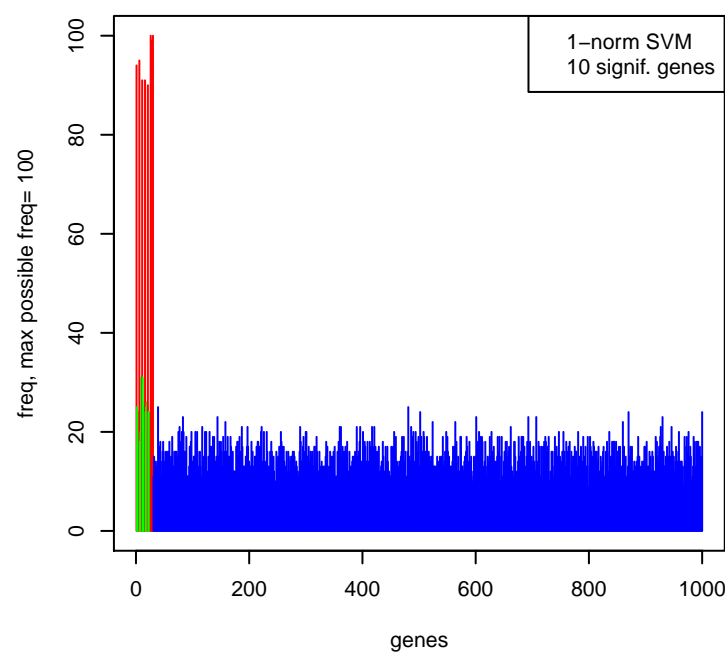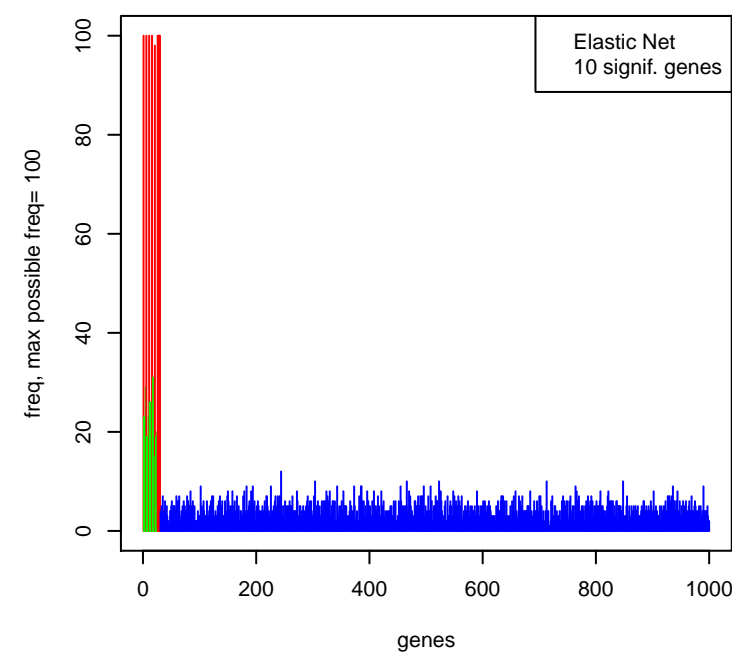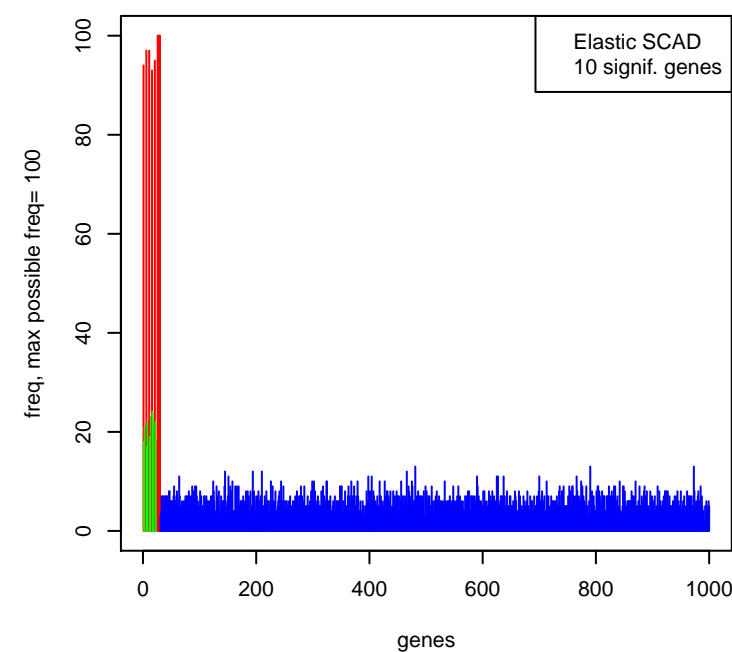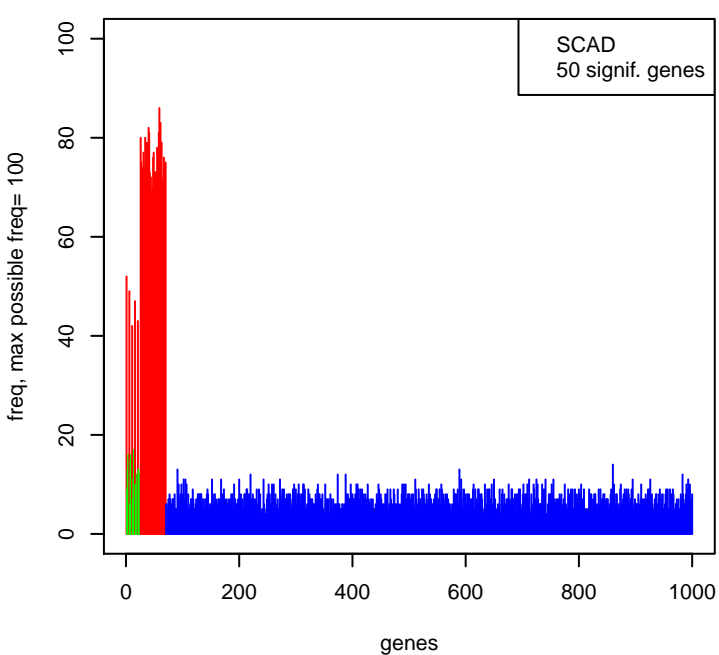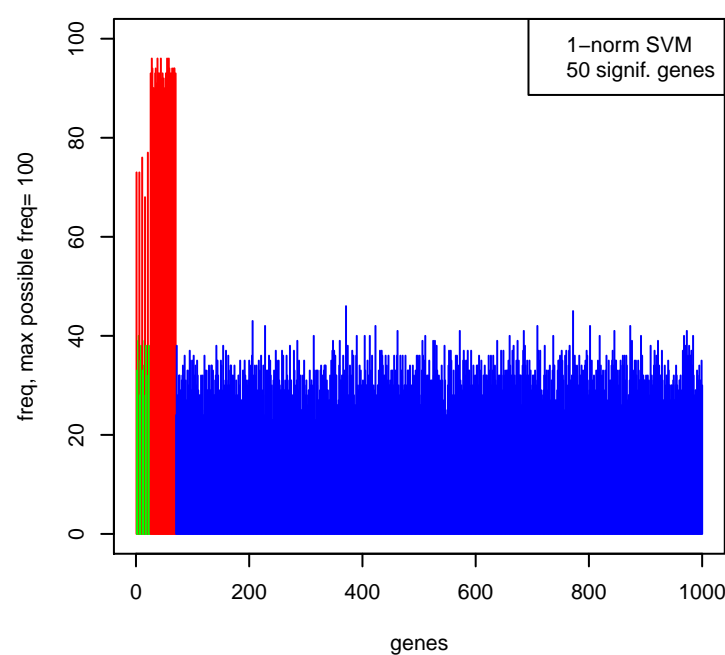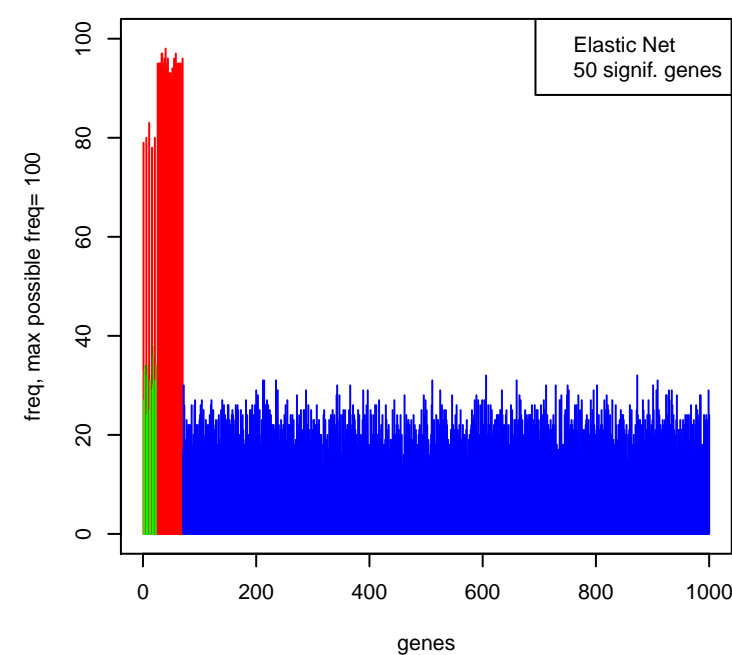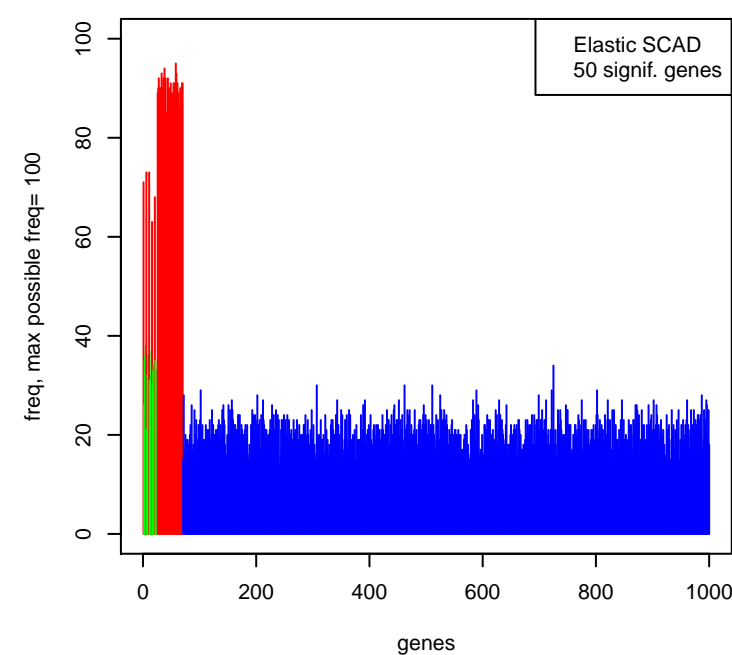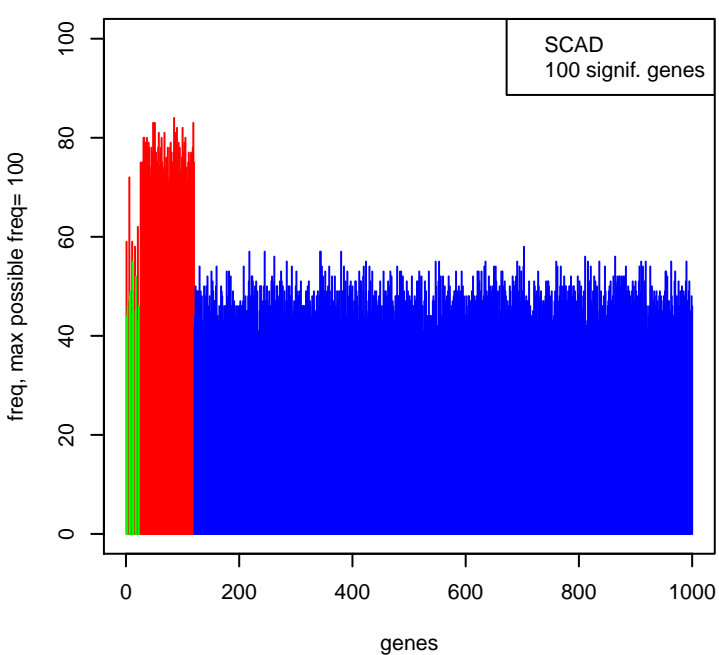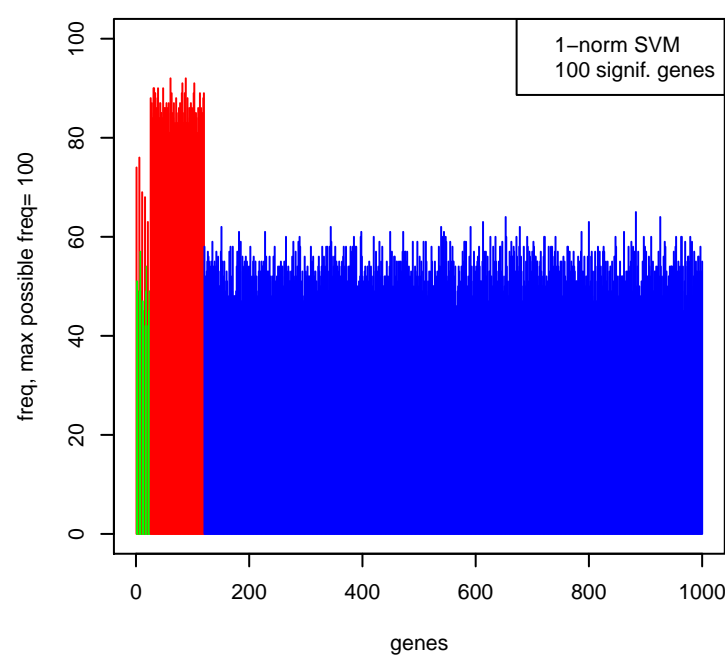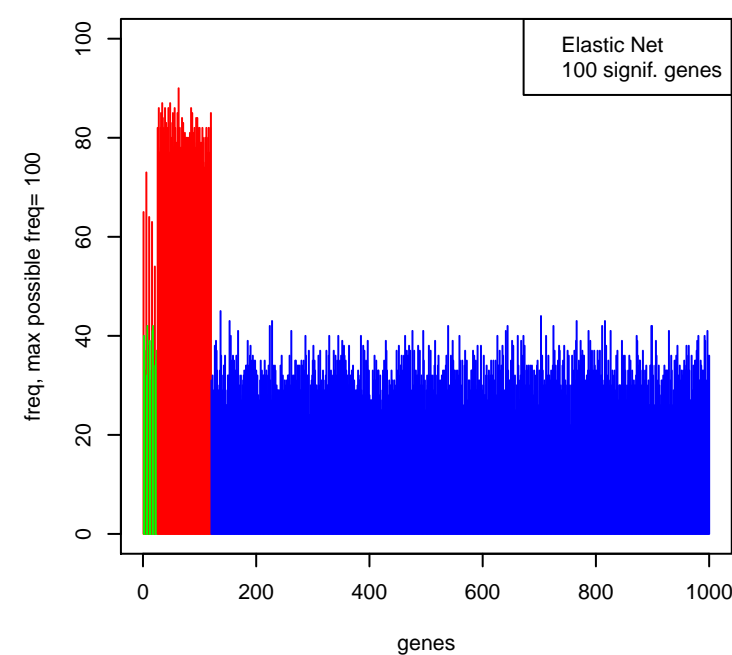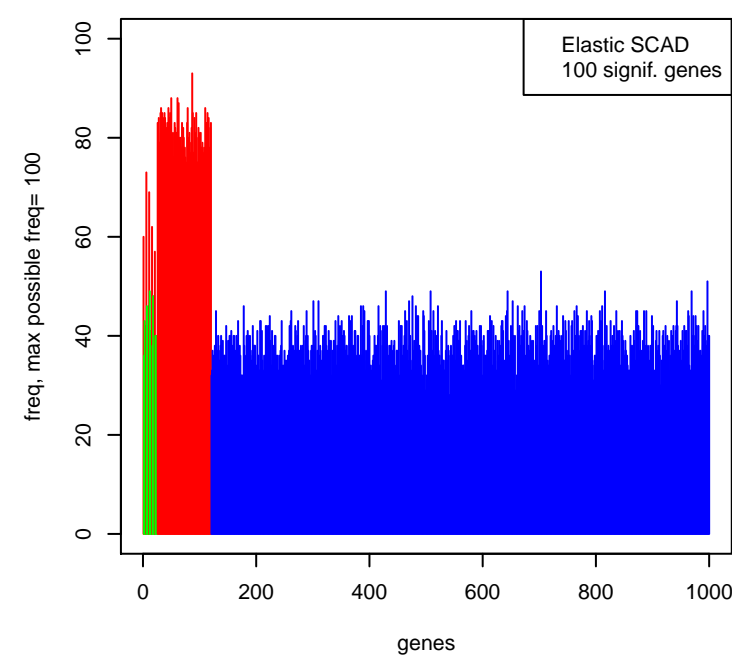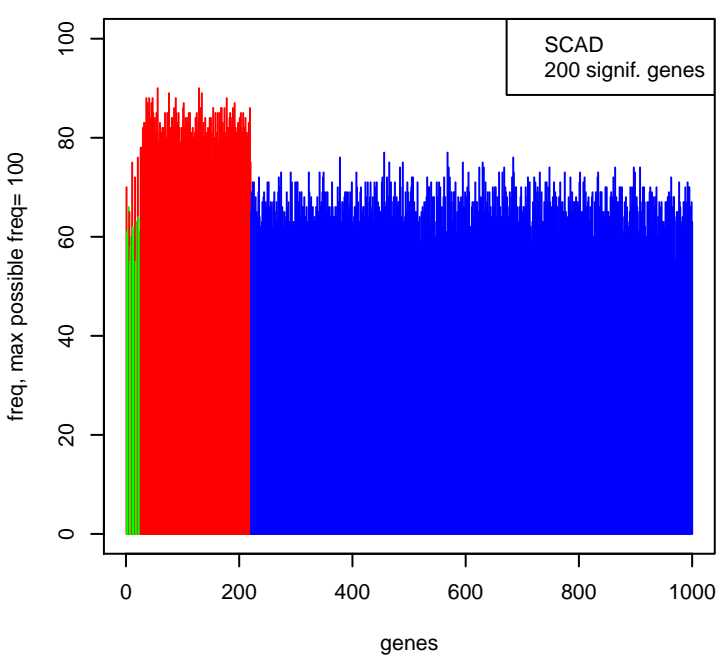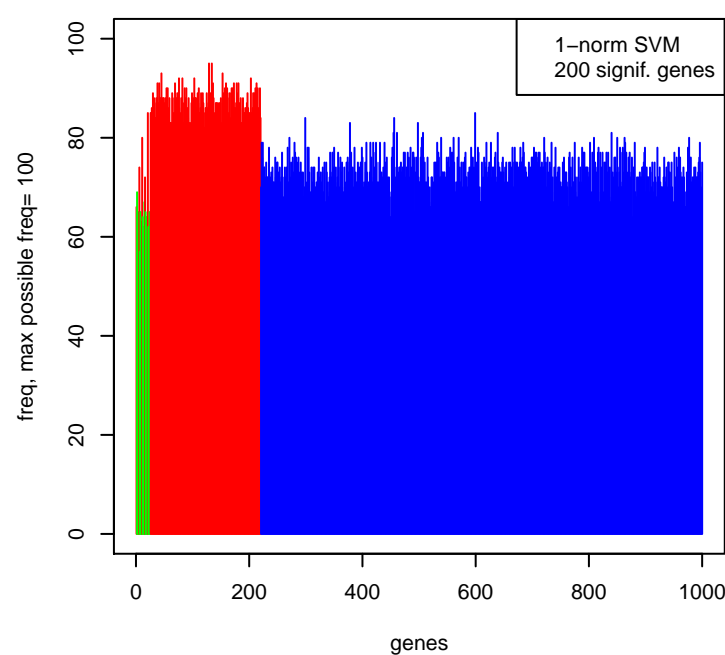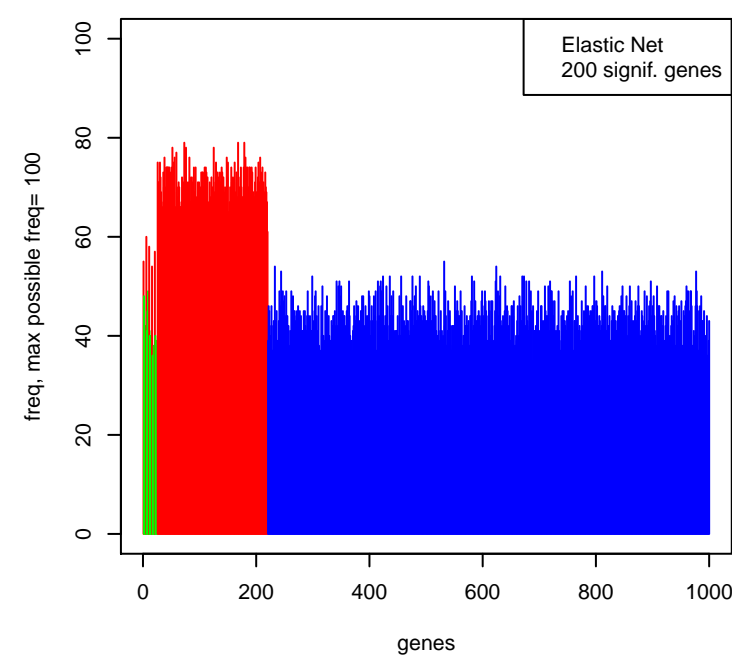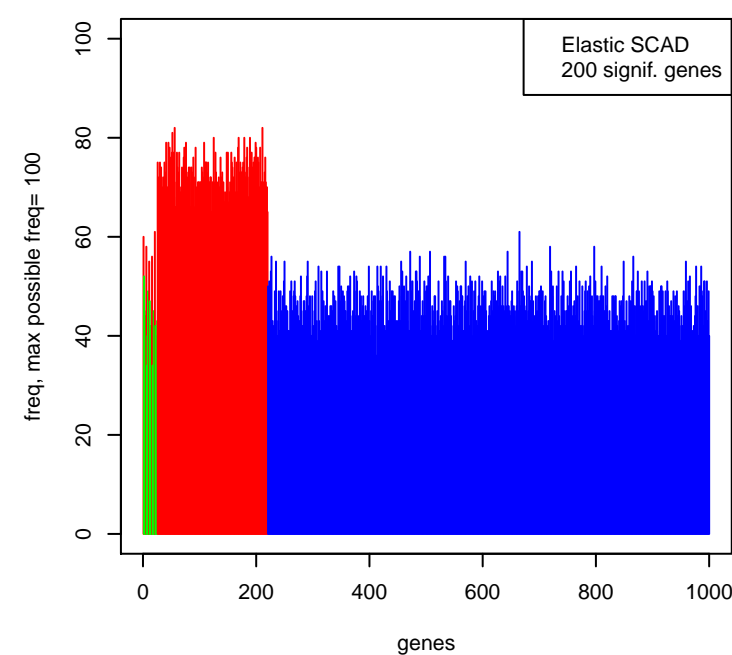

Supplement: Additional file 1 — Frequencies plot. Frequencies of selected features in the classifiers after 100 runs. In x-axis: features, y-axis: frequency of appearing of each features in classifiers after 100 runs. Features: true positives or non-zero (in red), zero features correlated with true positives (in green) and true negatives or zero (in blue). Algorithms from left to right: SCAD SVM, 1-norm (L1) SVM, Elastic Net SVM and Elastic SCAD SVM. Number of features: from top to bottom from very sparse till non-sparse models, r: 10, 50, 100, 200 out of 1000 features are relevant. [file 1471-2105-12-138-S1.PDF]
