# Supplementary material for: Elastic SCAD as a novel penalization method for SVM classification tasks in high-dimensional data
Source: BMC Bioinformatics. 2011 May 9;12:138. doi: 10.1186/1471-2105-12-138 (PMC3113938; doi:10.1186/1471-2105-12-138)
Supplement: Additional file 2 — Tables S1, S2, S3. Table S1: Mean frequency percentages for non-zero features in the classifier. Mean frequency percentages for non-zero features in the classifier (true positives) after 100 runs. Standard deviations in parentheses. Table S2: Mean frequency percentages for zero features, high correlated with non-zero features in the classifier. Mean frequency percentages for zero features, high correlated with non-zero features in the classifier after 100 runs. Standard deviations in parentheses. Table S3: Mean frequency percentages for independent non-zero features in the classifier (false positives). Mean frequency percentages for independent non-zero features in the classifier (false positives) after 100 runs. Standard deviations in parentheses. [file 1471-2105-12-138-S2.PDF]

Table 1. (Suppl.): Mean frequency percentages for non-zero features in the classifier (true positives) after 100 runs. Standard deviations in in parentheses.

FS method	r = 10	r = 50	r = 100	r = 200
L_1 SVM	$95.5_{(3.9)}$	$\underline{89.9}_{(2.0)}$	$\mathbf{84.5}_{(4.6)}$	$\mathbf{86.0}_{(4.3)}$
SCAD SVM	$96.2_{(4.2)}$	$71.5_{(9.6)}$	$75.1_{(4.8)}$	$\underline{81.2}_{(4.1)}$
Elastic Net SVM	$\mathbf{99.8}_{(0.6)}$	$\mathbf{92.4}_{(4.6)}$	$79.0_{(4.4)}$	$69.4_{(4.4)}$
Elastic SCAD SVM	$\underline{97.6}_{(2.8)}$	$87.2_{(6.6)}$	$\underline{79.4}_{(4.7)}$	$71.2_{(4.5)}$

In **bold** - maximum mean true positives in the classifier per simulation scenario; underline - the second best

Table 2. (Suppl.): Mean frequency percentages for zero features, high correlated with non-zero features in the classifier after 100 runs. Standard deviations in in parentheses.

FS method	r = 10	r = 50	r = 100	r = 200
L_1 SVM	$\mathbf{23.6}_{(3.7)}$	$\mathbf{34.1}_{(3.6)}$	$\mathbf{46.9}_{(3.2)}$	$\mathbf{62.5}_{(3.7)}$
SCAD SVM	$2.2_{(1.2)}$	$11.8_{(3.0)}$	$\underline{46.5}_{(3.5)}$	$\underline{58.6}_{(4.2)}$
Elastic Net SVM	$\underline{22.2}_{(4.4)}$	$30.5_{(4.0)}$	$35.6_{(4.0)}$	$39.2_{(4.8)}$
Elastic SCAD SVM	$19.5_{(2.3)}$	$\underline{32.0}_{(4.1)}$	$41.3_{(4.3)}$	$40.7_{(5.9)}$

In **bold** - maximum mean true positives in the classifier per simulation scenario; underline - the second best

Table 3. (Suppl.): Mean frequency percentages for independent non-zero features in the classifier (false positives) after 100 runs. Standard deviations in in parentheses.

FS method	r = 10	r = 50	r = 100	r = 200
L_1 SVM	$14.2_{(3.3)}$	$30.6_{(4.3)}$	$52.2_{(4.3)}$	$72.0_{(4.0)}$
SCAD SVM	$\mathbf{0.3}_{(0.5)}$	$\mathbf{6.5}_{(2.0)}$	$47.0_{(3.8)}$	$65.3_{(4.2)}$
Elastic Net SVM	$\underline{4.0}_{(1.9)}$	$21.0_{(3.9)}$	$\mathbf{31.3}_{(4.4)}$	$\mathbf{42.3}_{(4.6)}$
Elastic SCAD SVM	$5.7_{(3.8)}$	$\underline{19.6}_{(4.2)}$	$\underline{37.0}_{(4.5)}$	$\underline{44.7}_{(4.9)}$

In **bold** - minimum average number of false positives in classifier per simulation scenario; underline - the second best
